# Phytochemicals in *Daucus carota* and Their Health Benefits—Review Article

**DOI:** 10.3390/foods8090424

**Published:** 2019-09-19

**Authors:** Tanveer Ahmad, Maria Cawood, Qumer Iqbal, Agustín Ariño, Asmat Batool, Rana Muhammad Sabir Tariq, Muhammad Azam, Sajjad Akhtar

**Affiliations:** 1 Department of Horticulture, Ghazi University, Dera Ghazi Khan 32200, Pakistan; 2 Department of Plant Sciences, University of the Free State, Bloemfontein 9300, South Africa; CawoodME@ufs.ac.za; 3 Fiblast, LLC. 1602 Mizell Road, Tuskegee, AL 36083, USA; iqbalqumer@fiblast.com; 4 Veterinary Faculty, Instituto Agroalimentario de Aragón-IA2 (Universidad de Zaragoza—CITA), 50013 Zaragoza, Spain; aarino@unizar.es; 5 Institute of Horticultural Sciences, University of Agriculture, Faisalabad 38000, Pakistan; semee_uca@yahoo.com; 6 Department of Agriculture & Agribusiness Management, University of Karachi, Karachi 74000, Pakistan; sabir_tariq@yahoo.com; 7 National Institute of Food Science and Technology, University of Agriculture, Faisalabad 38000, Pakistan; muhammadazamfst@yahoo.com; 8 Department of Plant Sciences (Plant Breeding), University of the Free State, Bloemfontein 9300, South Africa; akhtarsajjad21@gmail.com

**Keywords:** carrot, phenolic compounds, carotenoids, polyacetylenes, ascorbic acid, human health

## Abstract

Carrots are a multi-nutritional food source. They are an important root vegetable, rich in natural bioactive compounds, which are recognised for their nutraceutical effects and health benefits. This review summarises the occurrence, biosynthesis, factors affecting concentration, and health benefits of phytochemicals found in *Daucus carota*. Two hundred and fifty-five articles including original research papers, books, and book chapters were analysed, of which one hundred and thirty articles (most relevant to the topic) were selected for writing the review article. The four types of phytochemicals found in carrots, namely phenolics, carotenoids, polyacetylenes, and ascorbic acid, were summarised. These chemicals aid in the risk reduction of cancer and cardiovascular diseases due to their antioxidant, anti-inflammatory, plasma lipid modification, and anti-tumour properties. Numerous factors influence the amount and type of phytochemicals present in carrots. Genotype (colour differences) plays an important role; high contents of α and β-carotene are present in orange carrots, lutein in yellow carrots, lycopene in red carrots, anthocyanins in the root of purple carrots, and phenolic compounds abound in black carrots. Carotenoids range between 3.2 mg/kg and 170 mg/kg, while vitamin C varies from 21 mg/kg to 775 mg/kg between cultivars. Growth temperatures of carrots influence the level of the sugars, carotenoids, and volatile compounds, so that growing in cool conditions results in a higher yield and quality of carrots, while higher temperatures would increase terpene synthesis, resulting in carrots with a bitter taste. It is worthwhile to investigate the cultivation of different genotypes under various environmental conditions to increase levels of phytochemicals and enhance the nutritional value of carrot, along with the valorisation of carrot by-products.

## 1. Introduction

Fruits and vegetables are rich sources of nutrients that contain phytochemicals (also known as bioactive compounds), which are recognised for their nutraceutical effects and health benefits [1]. The cultivated carrot (*Daucus carota* L.) is one of the most important vegetable plants in the world because of its high yield potential and use as fresh or processed product. With an annual world production (carrots and turnips) of >428 million tons and a total growing area of about 11.5 million hectares [2], carrots rank among the top 10 vegetable crops in the world [3]. They play a major role in human nutrition, because of their high dietary value and good storage attributes [4,5]. Phytochemicals contribute to the dietary value of carrots and comprise mainly four types; namely, phenolic compounds, carotenoids, polyacetylenes, and ascorbic acid. This review article comprehensively describes the occurrence, biosynthesis, factors affecting concentration, and health benefits of phytochemicals found in *Daucus carota*.

## 2. Methods

The literature for this review paper was retrieved from Google Scholar by using the following key words: occurrence of phenolics or phenols or phenolic acids, carotenoids, polyacetylenes, and ascorbic acid or vitamin C in carrot; biosynthesis of phenolics, or phenols or phenolic acids or hydroxycinnamic acids or chlorogenic acids, carotenoids, polyacetylenes, and ascorbic acid or vitamin C, in carrot; factors affecting the concentration of phenolics or phenols or phenolic acids, carotenoids, polyacetylenes, and ascorbic acid or vitamin C in carrot; nutritional importance or nutritional benefits of phenolics or phenols or phenolic acids, carotenoids, polyacetylenes, and ascorbic acid or vitamin C in carrot; health effects of carrot phenolics, carotenoids, polyacetylenes, and ascorbic acid/vitamin C after carrot consumption. The key words: structures of phenols or phenolic acids, carotenoids, polyacetylenes, and ascorbic acid or vitamin C in carrot, were searched in the NCBI website and redrawn in MS word using PNG format. Two hundred and fifty-five (255) articles including original research papers, books, and book chapters were downloaded, of which one hundred and thirty articles (130) most relevant to the topic were selected for writing the review article. The rejected research papers were too old or irrelevant. Literature was summarised according to the four types of phytochemicals found in carrots; namely, phenolics, carotenoids, polyacetylenes, and ascorbic acid. Under each phytochemical, the literature was summarised according to occurrence, biosynthesis, factors affecting concentrations, and resulting health benefits.

### The Carrot Plant

**Figure 1:**
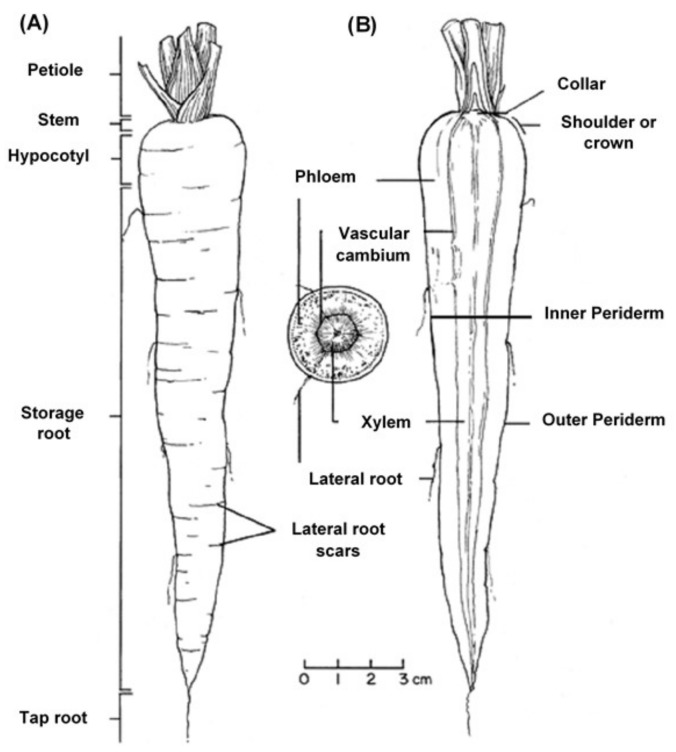
Carrot root anatomy: (**A**) longitudinal; (**B**) cross-section, showing the periderm, phloem, and xylem (www.carrotmuseum.co.uk).

The edible carrot, *Daucus carota*, is the most important root vegetable plant grown worldwide and it is part of the *Apiaceae* family [6]. The carrot consists mainly of two parts, the stem and the root, and most of the root consists of the peel (periderm), a pulpy outer cortex (phloem), and an inner core (xylem). Figure 1 shows the carrot root’s anatomy. Cultivated carrots have orange, reddish, purple, black, or yellow roots. The most commonly eaten part of the carrot plant is the root, though the stems and leaves are eaten as well, so the present review is in regard to the root, unless otherwise indicated.

## 3. Phenolic Compounds

Phenolic compounds constitute one of the most ubiquitous groups of plant metabolites and are an integral part of both human and animal diets. The role of polyphenols in the prevention of degenerative diseases, like cancer, cardiovascular diseases, and neurodegenerative diseases has been reported. Interest in food phenolics has increased greatly over the past two decades, owing to their antioxidant capacity and their function as a defence against oxidative stress caused by excess reactive oxygen species [7].

**Figure 2:**
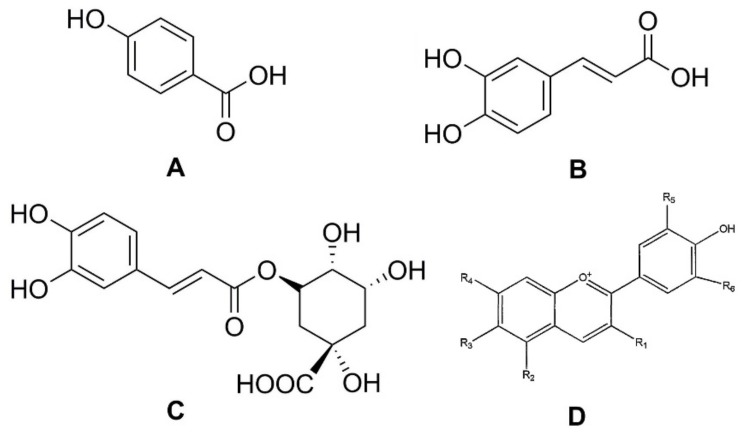
Structures of phenolic acids: (**A**) *p*-hydroxybenzoic acid; (**B**) caffeic acid; (**C**) chlorogenic acid; and (**D**) the basic chemical structure of anthocyanins.

Phenolic compounds are secondary plant metabolites, mainly composed of an aromatic ring bearing one or more hydroxyl groups, playing a crucial role in counteracting various type of stress (ultraviolet irradiation, aggression by pathogens, parasites, and plant predators), and contributing to the organoleptic properties of plants and plant-derived food [8,9,10,11]. Phenolic compounds can be divided into different subgroups, such as phenolic acids, flavonoids, tannins, lignans, stilbenoids, and curcuminoids. It has been reported that carrots are rich in phenolic acids, such as *p*-hydroxybenzoic, caffeic, and chlorogenic, as well as in anthocyanins, a class of flavonoids (Figure 2) [12].

Isocoumarins and phenolic acids are the potentially bitter compounds found in carrot peels. Czepa and Hofmann [13] reported that the bitter taste in carrots is caused by terpenoids and water-soluble phenolics. Therefore, their presences can be used as biological markers to assess the quality of fruits and vegetables during postharvest operations [14].

### 3.1. Occurrence of Phenolic Compounds

Phenolic compounds are present in high concentrations in the root periderm tissues of carrots. Carrot roots contain hydroxycinnamic acids and derivatives [15]. Sharma et al. [14] reported that chlorogenic acid was the main hydroxycinnamic acid identified in different carrot tissues, accounting for 42.2% to 61.8% of total phenolics. The concentrations of phenolic compounds in different carrot root tissues decrease from the peel (periderm) to the xylem (Figure 1). The peel is only 11% of the total fresh weight of carrot but contains 54.1% of the total phenolic compounds, followed by the phloem (39.5%) and xylem (6.4%). However, the concentration depends on the cultivar, the extraction method, the manner in which the results are expressed, and the post-harvest and processing circumstances [15,16]. Carrots of different colours displayed a high variation in antioxidant properties [17]. The results consistently indicated that among different carrot colours, purple exhibited the highest antioxidant capacity due to its higher phenolic compound concentration [4]. It has been reported that carrot contains 27 ± 1.7 µg/g gallic acid equivalents of phenolic compounds, as determined with Folin–Ciocalteu reagent [18].

### 3.2. Biosynthesis of Phenolic Compounds

Phenolic compounds are formed biosynthetically from either the shikimic acid pathway or the acetate pathway. Plant-derived phenylpropanoids, with a C6–C3 skeleton, compose the largest group of secondary metabolites produced by higher plants. They are parent molecules for the biosynthesis of numerous structurally and functionally diverse plant polyphenols (simple phenolic acids and esters, glycosylated derivatives of primary phenylpropanoids, flavonoids, isoflavonoids, stilbenes, coumarins, curcuminoids, lignans, etc.), which play multiple essential roles in plant physiology [19]. The phenylpropanoid, chlorogenic acid, a derivative of hydroxycinnamic acid, was reported to be the most common phenolic compound in carrots [14] and it is biosynthesised via the shikimic acid pathway [20,21].

**Figure 3:**
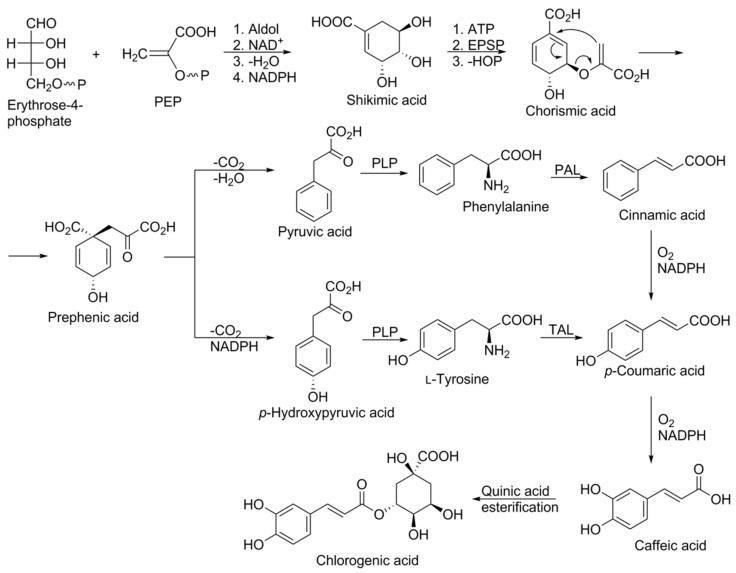
Schematic representation of the biosynthesis of chlorogenic acid via the shikimic acid pathway. Abbreviations: Aldol: aldol condensation reaction; ATP: adenosine triphosphate; EPSP: 5-enolpyruvylshikimate-3-phosphate synthase; NAD and NADH: nicotinamide adenine dinucleotide reductase and oxidase; NADPH: nicotinamide adenine dinucleotide phosphate-oxidase; PAL: phenylalanine ammonia lyase; TAL: tyrosine ammonia lyase; PLP: pyridoxal phosphate.

The synthesis starts with an aldol condensation reaction between phosphoenol pyruvate (PEP) and erythrose 4-phosphate, and culminates through various stages in the formation of prephenic acid. Prephenic acid serves as a branch point in the pathway and rearranges either via decarboxylative 1,4-dehydration to yield phenylpyruvic acid, or decarboxalative 1,4-dehydrogenation to yield *p*-hydroxyphenylpyruvic acid. Subsequent transamination with pyridoxal phosphate yields the amino acids phenylalanine and tyrosine (C6–C3 structures), respectively. Phenylalanine ammonia lyase (PAL) and tyrosine ammonia lyase (TAL) are key enzymes of the phenylpropanoid pathway which catalyse the conversion of phenylalanine to cinnamic acid and of tyrosine to 4-hydroxycinnamic acid (p-coumaric acid), respectively. Cinnamic acid is further transformed, through the catalytic action of different enzymes; e.g., cinnamate 2-hydroxylase followed by 4-coumaroyl CoA-ligase to produce 4-hydroxycinnamic acid (p-coumaric acid), which is converted into caffeic acid and finally chlorogenic acid (Figure 3). 4-Coumaroyl CoA probably represents the most important branch point within the central phenylpropanoid biosynthesis in plants [20,22,23].

### 3.3. Factors Affecting Phenolics Concentration

Phenolic compounds are affected by multiple factors, such as the cultivar, storage conditions and temperature, fertilizer application, processing procedures, and various biotic and abiotic stress factors [24].

#### 3.3.1. Cultivar

Alasalvar et al. [16] reported that fresh purple genotypes have 2.9 times higher phenolic concentrations (102 ± 3.8 mg/100 g) than orange genotypes (34.8 ± 1.9 mg/100 g).

#### 3.3.2. Fertilizer Application

The application of different doses of fertiliser and fertiliser varieties in different growth systems (conventional and organic) and in different locations significantly affected phenolic content [25]. For example, a deficiency of boron increases the accumulation of phenolics, and the application of nitrogen fertiliser could change phenolic concentration in carrot roots [26].

#### 3.3.3. Storage Conditions and Temperature

Storage conditions and temperature affect the concentration of phenolic compounds, particularly that of chlorogenic acid. In a recent study by Kamiloglu et al. [27] with black carrots, it was found that after 20 weeks of storage, the preserved anthocyanins in samples stored at 4 °C (53.4%–81.0%) were higher than samples stored at 25 °C (7.8%–69.3%). Simões et al. [28] investigated the effect of controlled atmosphere on baby carrots and reported that controlled atmosphere of 5 kPa O_2_ and 5 kPa CO_2_ significantly increased chlorogenic acid content. In general, plants subjected to postharvest abiotic stresses synthesize secondary metabolites, such as phenolic compounds. Wounding stress, water loss, peel removal, and UV light affect the biosynthesis of phenolic compounds in carrots [29,30,31,32,33]. Wounding stress induced an increase of ∼287% in total phenolic content (PC) in carrots stored for 48 h at 20 °C, while this increase was higher (∼349%) in the wounded tissue treated with low-oxygen stress [34]. Wounding stress (23.5 cm^2^/g) produced approximately 2.5 times more soluble phenols in carrots than in undamaged carrots. It mostly stimulates the synthesis of chlorogenic acid (5-CQA) and 3,5-dicaffeoylquinic acid, which enhances the antioxidant capacity of carrots [35].

#### 3.3.4. Processing Procedures

Ma et al. [36] studied the effects of different processing units on fresh carrot juice and concluded that blanching and enzyme liquefaction can increase total phenolic content, while pasteurization decreases total phenolic content compared to fresh carrot juice. Another study reported that un-blanched frozen and blanched (soaking carrots in water at 95 °C for three minutes) frozen treatments non-significantly affected phenolic concentration in carrots, even after seven days of storage at 4 °C [37]. The processing of black carrots into jam and marmalade led to a decrease in total phenolic content of 89.2% to 90.5%, antioxidant capacity by 83.3% to 91.3%, and phenolic acids by 49.5 to 96.7%. The reasons are that cell structures are destroyed during processing of black carrots into jam and raw material is exposed to both enzymatic and non-enzymatic oxidation, resulting in the loss of phenolic compounds [27].

### 3.4. The Health Benefits of Phenolic Compounds

It has been estimated that the average dietary intake of polyphenols is 1058 mg per day for males and 780 mg per day for females, comprised as follows: 50% hydroxycinnamic acids, 20% to 25% flavonoids, and 1% anthocyanins [38]. The dietary intake of phenolic compounds has been related to favourable health impacts, particularly due to their antioxidant activity, such as anti-aging, anti-inflammatory, and antiproliferative effects. Additionally, these compounds contribute to the maintenance of normal blood glucose and cholesterol levels, as well as to the normal functioning of the nervous system. Due to their antioxidant properties, the risk of cardiovascular diseases is minimised, and polyphenols possess anti-ageing properties, as well as anti-carcinogenic properties, by functioning as free-radical scavengers [39]. Polyphenols also potentially protect against diabetes and Alzheimer’s disease [38]. They enhance bile secretion, decrease cholesterol and lipid levels in the blood, and exhibit antimicrobial properties towards *Staphylococcus aureus* [40].

Preclinical and epidemiological studies suggest that polyphenols might be helpful in reversing neurodegenerative pathogenic actions and ageing in neurocognitive development. However, there is no evidence for the role of polyphenols in the improvement of neurological health. Their potential roles are due to their capability to interrelate with intracellular neuronal and glial signalling, affect peripheral and cerebrovascular blood flow, and lessen neural injury and damage caused by neurotoxins and the inflammation of neurons [41]. Carrot genotypes exhibited diverse antioxidant capacities; in low rainfall seasons, the white genotypes contain higher quantities of phenolic compounds [4].

The anthocyanins of black carrot are effective for the risk reduction of different types of cancer. The growth of HT-29 and HL-60 cancer cells were inhibited by 80% when 2.0 mg/mL of the lyophilised powder of aqueous black carrot was ingested [42,43,44]. Ethanol extracts of black carrot anthocyanins were used in the treatment of human colon, breast, and prostate cancers and were found to have antioxidant and anti-proliferative properties against different cancer cell lines [45]. Cancer types resistant to chemotherapy can be treated using black carrot extract alone or together with anticancer drugs.

The bioactive compounds of black carrot were also found to reduce cardiovascular diseases by decreasing the blood cholesterol and glucose levels; additionally, cholesterol production in liver is reduced by the inhibition of 3-hydroxy-3-methylglutaryl-coenzyme A reductase activity. Lymphocyte activation, the inhibition of cell proliferation, anti-inflammatory effects, a reduction of the body mass index, a reduction in triglyceride level and blood pressure, and a reduction in the binding activity of bile acid are other distinguishable properties of bioactive compounds of black carrot that help in the prevention of cardiovascular diseases [46]. Phenolic compounds of black carrots are also helpful in reducing the risk of diabetes [44].

## 4. Carotenoids

**Figure 4:**
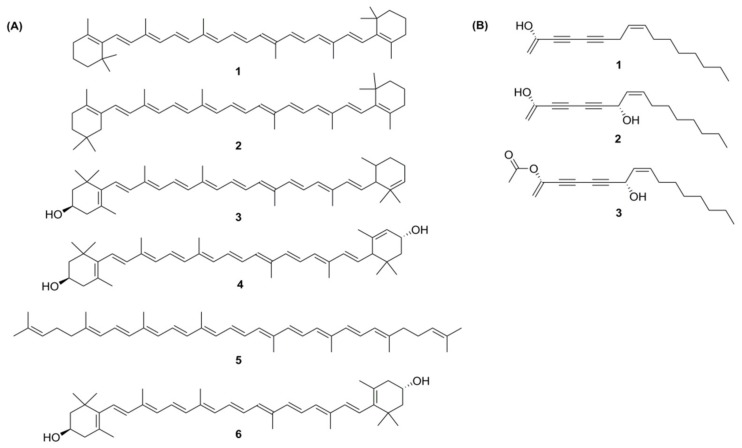
(**A**) Structures of carotenoids: (1) α-carotene; (2) β-carotene; (3) β-cryptoxanthin; (4) lutein; (5) lycopene; (6) zeaxanthin; (**B**) Structures of the polyacetylenes: (1) falcarinol; (2) falcarindiol; (3) falcarindiol-3-acetate.

Carotenoids are a group of isoprenoid molecules present in all photosynthetic plants, including carrots. Some non-photosynthetic fungi and bacteria also possess carotenoids. Carotenoids are acyclic or have five or six C rings on one or both ends of the molecule [14]. Several conjugated double bonds of a polyene chain that function as a chromophore are responsible for the yellow, orange, and red colours of carotenoids [47,48]. There are two types of carotenoids present in carrot; namely, carotenes and xanthophylls. The major carotenoids (Figure 4A) in carrot roots are β-carotene (75%); α-carotene (23%); lutein (1.9%); and β-cryptoxanthin, lycopene, and zeaxanthin [49].

### 4.1. Occurrence of Carotenoids

Carotenoids are named after carrot, because carrot accumulates an enormous number of carotenoids in its roots. Orange and purple carrots have higher concentrations of carotenoids in the phloem than in the xylem [50]. Beta-carotene makes up 80% of total carotenoids contained in domestic carrot roots [51]. Generally, a carrot contains 16 to 38 mg/100 g carotenoids [52].

### 4.2. The Biosynthesis of Carotenoids

Carotenoids are formed in plastids from isoprenoid precursors via the methylerythritol 4-phosphate (MEP) pathway [53]. In the first step, 15-cis-phytoene (colourless carotenoid) is produced by the catalytic action of phytoene synthase (PSY). This compound is desaturated, isomerised, and converted into reddish all-trans lycopene via the catalytic actions of phytoene desaturase (PDS), 15-cis-γ-carotene isomerase (ZISO), γ-carotene desaturase (ZDS), and carotenoid (pro-lycopene) isomerase (CRTISO).

**Figure 5:**
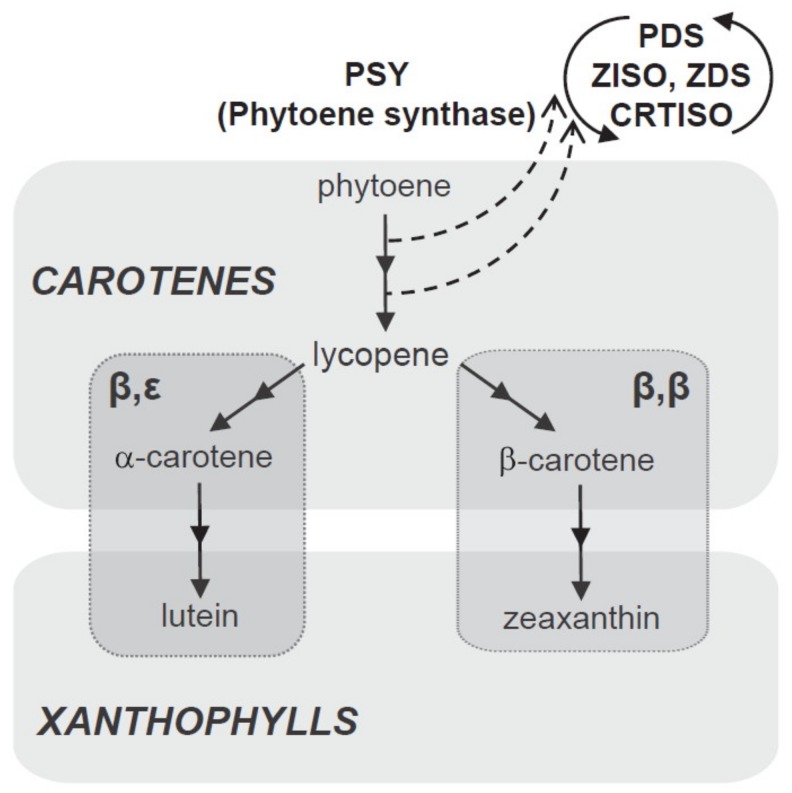
Schematic biosynthetic pathway for carotenoids.

During cyclization of lycopene, the pathway splits into two branches to yield two orange carotenes; namely, beta (β) and epsilom (ε). Beta-carotene (with two β-rings on two ends of the lycopene molecule) is formed via lycopene β-cyclase (LCYB) and α-carotenes (one β-ring on one end and one ε-ring on the other) via lycopene β-cyclase (LCYE). Zeaxanthin is produced by the hydroxylation of β-carotene by carotenoid β-hydroxylase (CHYB) enzymes, particularly of the nonheme diiron (BCH) type; meanwhile, yellowish xanthophyll lutein is formed by hydroxylation of α-carotene catalysed by β and ε-hydroxylase (CHYB and CHYE) enzymes, primarily of the cytochrome P450 (CYP97) type [48,49,54]. A schematic biosynthetic pathway for carotenoids is depicted in Figure 5.

### 4.3. Factors Affecting Carotenoids’ Concentrations

Carotenoids are influenced by two main factors; i.e., inherited characteristics and the environment [17,55,56].

#### 4.3.1. Cultivar

A seven to eleven-fold difference in β-carotene concentration was observed in cultivars with different genetic makeups [57]. There is controversy in the literature about the highest number of carotenoids present in different carrot genotypes. In previous studies, Alasalvar et al. [58] found 2.3 times more β-carotenes in purple carrots than orange varieties. Similar results were presented by Seljåsen et al. [57]. However, according to recent studies, higher contents of α and β-carotene are present in orange carrots, lutein in yellow carrots, lycopene in red carrots, anthocyanins in the roots of purple carrots, and phenolic compounds in black carrots [44,59,60,61]. Nicolle et al. [62] studied the effect of genetic variability on carotenoids in carrots of different colours (genotypes) and found that the range of carotenoids in yellow and purple carrots is 469 to 605 μg/100 g, while 10 times more carotenoids are present in orange carrots. The highest carotenoids’ content, particularly β-carotene (170 mg/kg), is present in dark orange carrots, whereas purple carrots have the lowest β-carotene content (3.2 mg/kg). Suggestions for retaining carotenoid concentrations include the usage of cultivars known to have higher ranges of the useful compounds, and those which might be more suited to the local weather and geographical location. Gene expression partly describes the differences in carotenoids’ accumulation in the secondary phloem and xylem of the fleshy roots of carrots [50].

#### 4.3.2. Environment

Environmental conditions during growth and packaging alter the level of carotenoids, sugars, and volatiles [55,63]; however, results may vary when research is conducted under different conditions. Other researchers, Martín-Diana et al. [64] and Rico et al. [65], emphasise that crops grown in sandy soil tend to build up fewer provitamin A carotenoids than those grown in clay soils.

#### 4.3.3. Storage Conditions and Temperature

Retail storage of carrots often takes place at a temperature range of 18 to 22 °C. Carrots can be subjected to these temperatures for a few days. Storage’s effect on carrot β-carotene is inconsistent based on different temperature levels. According to Imsic et al. [66], α and β-carotene concentrations increased up to 35% and 25% after three days of storage and up to 42% and 34% after ten days of storage in Nantes carrots stored at 2 °C and 90% relative humidity. Significant increases in β-carotene were observed in both Nevis and Kingston cultivars stored at 20 °C for seven days. Longer storage periods of 21 days at 20 °C have a negative effect on α and β-carotene [66]. After four weeks of storage, β-carotene was enhanced from 8% to 23% at 4 °C, compared to the levels at harvesting time [57,67]. Negi and Roy [68] reported that β-carotene contents were reduced after eight days of storage at different temperatures, by 46% (7.5 to 8.5 °C), 51% (17 to 21 °C), and 70% (22 to 37.5 °C). Some studies also evidenced slight variations in α or β-carotene when carrots were stored at 0 °C, even for six months [69].

### 4.4. Health Benefits of Carotenoids

The dietary intake of carotenoids, especially vitamin A, has been related to the protection of DNA, proteins, and lipids from oxidative damage; as well as to the maintenance of the normal function of the immune system, normal skin, normal mucosal membranes, and normal vision [49,52,70,71]. Digested purple carrot extract when passed through the colon mucosal cells, decreases oxidative DNA damage by 20.7%, protecting colon cells against reactive oxygen species stress [72].

Beta-carotene, which is present in purple and orange carrots, is the most widely studied carotenoid so far, due to its significance in medical science. Dietary provitamin A carotenoids derived from plants are a major source of our vitamin A needs, and bioconversion to retinol may account for one-third of total retinol intake in developed countries. Vitamin A is essential for normal organogenesis, immune functions, tissue differentiation, and eyesight [71]. Alpha-carotene, β-carotene, and β-cryptoxanthin obtained from carrot consumption are the carotenes that are converted into retinol in the human body.

Lutein from yellow carrot and its isomer, zeaxanthin, both accumulate in the centre of the retina (also known as the macula) of the eye. These are the only carotenoids that pass through the retinal barrier and form the macula in the eye. The macula enhances eyesight through its light-filtering characteristics. They are also powerful antioxidants and essential for healthy eyes. They protect eyes from diseases by absorbing harmful blue light that enters the eye. Lutein is also the most dominant carotenoid in brain tissue and the predominant carotenoid in the developing primate brain and retina. The amount of lutein is twice as much in paediatric brains than in adult brains, indicating its role in neural growth, and it may play a role in biological functions, including anti-oxidation, anti-inflammation, and in structural activity. It shields neural tissue, especially during infancy when the retina and brain are continuously in a state of change after birth. In adults, it is linked to cognitive health, and its supplementation enhances cognition. High ingestion (near 6 mg per day) of lutein is associated with low risk of muscular degeneration during old age, although actual intake of lutein varies between 1 and 2 mg per day in adults. It can also prevent the production of harmful free radicals, such as reactive oxygen species, via physical or chemical quenching of singlet oxygen [73,74,75].

## 5. Polyacetylenes

Polyacetylenes are a prominent group of non-volatile bioactive phytochemicals that comprise at least two conjugated triple C–C bonds. Plants of the *Apiaceae* family (to which the carrot belongs) contain aliphatic C_17_-polyacetylenes of the falcarinol type [76]. Recent studies on the biological activity of polyacetylenes have indicated their potential to improve human health due to anticancerous, antifungal, antibacterial, anti-inflammatory, and serotogenic effects. These findings suggest targeting vegetables with elevated levels of bisacetylenic oxylipins, such as falcarinol; and due to the abundant availability, high diversity of cultivars, worldwide experience, and high agricultural yields, carrot (*Daucus carota* L.) genotypes are a very promising target vegetable [3].

From more than 1400 polyacetylenes identified in plants, 12 polyacetylenes were isolated from carrot. Out of the twelve, falcarinol, falcarindiol, and falcarindiol-3-acetate are essential polyacetylenes predominately found in carrot roots (Figure 4B). The other nine polyacetylenes that are isolated from carrot are: (E)-isofalcarinolone, falcarindiol-8-acetate, 1,2-dihydrofalcarindiol-3-acetate, (E)-falcarindiolone-8-acetate, (E)-falcarindiolone-9-acetate, 1,2-dihydrofalcarindiol, (E)-1-methoxy-falcarindiolone-8-acetate, (E)-1-methoxy-falcarindiolone-9-acetate, and panaxydiol [3,77].

### 5.1. The Occurrence of Polyacetylenes

The polyacetylenes are mainly present in the pericyclic parenchyma of the root and phloem near the secondary cambium [78,79]. Falcarinol is uniformly distributed in the carrot peel [80] and it is allocated to all parts of carrot root, while falcarindiol and falcarindiol-3-acetate are more abundant inside the higher and outer segments, respectively [13,79].

### 5.2. Biosynthesis of Polyacetylenes

**Figure 6:**
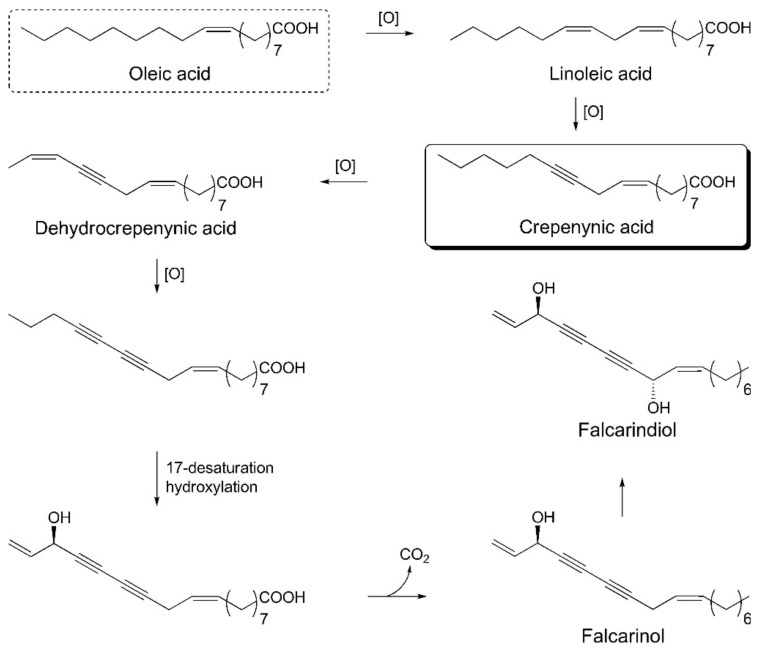
Schematic biosynthetic pathway for falcarinol type polyacetylenes.

The crepenynate pathway is involved in the biosynthesis of falcarinol-type polyacetylenes. Acetyl-CoA and malonyl-CoA react in the presence of fatty acid synthase and Δ^9^-desaturase enzymes and are converted into oleic acid. Oleic acid undergoes dehydrogenation to C_18_-acetylenes—linoleic acid, crepenynic acid, and dehydrocrepenynic acid—by the catalytic action of Δ^12^-desaturase, Δ^12^-acetylenase, and Δ^14^-desaturase enzymes, respectively. Dehydrocrepenynic acid is further transformed into C_17_-acetylenes in the presence of Δ^14^-acetylenase through β-oxidation [3,81]. A schematic biosynthetic pathway for falcarinol type polyacetylenes is depicted in Figure 6.

### 5.3. Factors Affecting Polyacetylenes’ Concentration

The amount of falcarinol-type polyacetylenes in carrots is significantly affected by cultivar, geographic area (location), root size, harvesting time [79], storage conditions [82], industrial processing [83], and biotic and abiotic stresses during the growing period and postharvest practices [13,56].

#### 5.3.1. Cultivar and Location

Cultivars affect the polyacetylenes in carrot; for instance, cultivated orange carrots contain falcarinol, falcarindiol, and falcarindiol-3-acetate in the range of 16 to 84 mg/kg, 8 to 40 mg/kg, and 8 to 27 mg/kg of fresh weight, respectively [3,13,84]. The concentration of falcarindiol polyacetylenes was observed within the range of 7 to 40.6 mg/kg of fresh weight when 27 different carrot cultivars were grown and harvested under the same growing conditions. The concentration of polyacetylenes found in the carrot may be 10–20 times more in wild type of carrots than domesticated carrots [3,85].

In another experiment, Kidmose et al. [56] studied the effect of six carrot genotypes on polyacetylenes’ concentration by growing them in two different locations and found significant variations in the range of falcarinol (0.4 to 1.6 mg/100 g), falcarindiol (1.9 to 5.4 mg/100 g), and falcarindiol-3-acetate (0.9 to 1.9 mg/100 g) in fresh weights. Kjellenberg et al. [86] reported the influence of the chemical composition of the soil on the concentrations of falcarinol-type polyacetylenes in carrots. Carrots grown in soils generally low in available phosphorus exhibited higher levels of falcarindiol if the soil was also low in available magnesium and calcium.

#### 5.3.2. Root Size

It is reported that the concentrations of falcarindiol and falcarindiol-3-acetate decrease by increasing the root size of carrot, while the concentration of falcarinol is independent of root size. That is because falcarinol is mostly present in the phloem, while falcarindiol and falcarindiol-3-acetate are present in the root’s periderm [56].

#### 5.3.3. Harvesting Time

Kjellenberg [87] indicated that the falcarinol level was slightly enriched after harvesting (with a short storage span), ultimately reaching a stabilised stage. The concentration of falcarindiol and falcarindiol-3-acetate reduced during early harvesting and increased during late harvesting dates, while falcarinol concentration did not change significantly. Similar effects were observed during storage [79].

#### 5.3.4. Processing and Storage

High concentrations of falcarinol, falcarindiol, and falcarindiol-3-acetate were documented in whole carrots that were refrigerated for four months at 1 °C. This indicates that polyacetylenes were produced during postharvest storage or there was little degradation in intact carrots after cold storage [56]. According to Rawson et al. [88], polyacetylene concentrations in carrot disks decreased at low temperatures (50 to 60 °C) and increased at high temperatures (70 to 100 °C), particularly the concentration of falcarinol. High pressure-temperature (HPT) processing enhances the retention of polyacetylenes in carrots. The highest combination which gave the maximum retention of falcarinol was 400 MPa, at 50 and 60 °C for 10 min; for falcarindiol it was 400 MPa, at 50 °C for 10 min; and for falcarindiol-3-acetate, it was 400 MPa, at 50 °C for 10 min.

Blanching and rapid freezing increased the retention rate of polyacetylenes in carrots during storage in cool conditions [89,90]. Kidmose et al. [56] found an increase in falcarinol contents in frozen carrots that were blanched before freezing. Similarly, ultrasound and blanching pre-treatments affect the concentration of polyacetylenes in freeze-dried and hot-air-dried carrots. An ultrasound followed by hot-air drying results in a higher retention of polyacetylenes in dried carrot discs than blanching. Moreover, freeze-dried samples exhibit a better retention of polyacetylenes than those of hot-air-dried samples [91]. Koidis et al. [92] stated that peeling also affects the retention of polyacetylenes in carrots. Peeled carrots have a higher amount of polyacetylenes, but when washed after cutting, the contents decrease substantially due to leakage.

### 5.4. Health Benefits of Polyacetylenes

Polyacetylenes are reported to have health promoting traits, and in vitro data suggest that plant extracts containing falcarinol type polyacetylenes have anti-cancer and anti-inflammatory actions. Polyacetylenes are extremely cytotoxic against several cancer cell lines and have revealed antifungal, anti-inflammatory, and anti-platelet aggregatory characteristics [93]. Purup et al. [94] suggested that the hydroxyl group (–OH) at C_3_ may account for these activities. Polyacetylenes of carrots are associated with health benefits [95], and more specifically, it was found that the falcarinol-type polyacetylenes from carrot shields against cancer [80]. More recently, Tan et al. [96] documented that C_17_-polyacetylenes inhibit the breast cancer resistance protein BCRP/ABCG2 when used as a multidrug resistance reversal agent. Kjellenberg et al. [97] studied polyacetylenes in fresh and stored carrots and reported that falcarinol activates mammalian cell differentiation, but also showed toxic effects against human cancer cells and the possibility of allergic inflammation of the skin. Falcarinol and falcarindiol may be used as antidiabetic agents in the treatment of diabetes due to their ability to arouse basal or insulin-dependent glucose absorption in adipocytes and porcine myotube cell cultures based on different doses [98].

Zaini et al. [99] studied the role of the bioactive chemicals of carrot juice extract in the treatment of leukaemia and concluded that carrot juice extracts can cause apoptosis, resulting in the cell cycle arrest of cells affected by leukaemia. That is why carrot juice extracts can be the best reservoir of bioactive compounds suitable for treatment of leukaemia. In another study, it was suggested that instead of beta-carotenes or lutein, polyacetylenes are the bioactive compounds from carrot that would be effective in the treatment of leukaemia [100]. Polyacetylenes of purple carrots are involved in anti-inflammatory bioactivity in humans. These polyacetylenes enhance cell proliferation at lower concentrations. Falcarinol is the most bioactive polyacetylene, inducing epithelial cell proliferation at the low concentration range of 0.004 to 0.4 μM [101].

## 6. Ascorbic Acid

**Figure 7:**
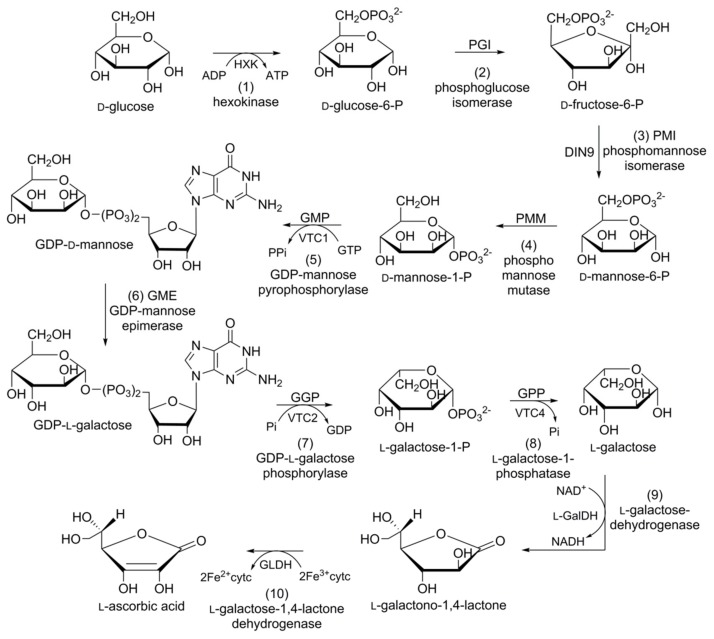
Schematic representation of the biosynthetic pathway of ascorbic acid in carrot: (1) hexokinase; (2) phosphoglucose isomerase; (3) phosphomannose isomerase; (4) phosphomannose mutase; (5) guanosine diphosphate (GDP)-mannose pyrophosphorylase; (6) GDP-mannose epimerase; (7) GDP-l-galactose phosphorylase; (8) l-galactose-phosphatase; (9) l-galactose-dehydrogenase; (10) l-galactose-1,4-lactone dehydrogenase.

l-ascorbic acid or vitamin C (Figure 7) is one of the most abundant water-soluble low molecular weight antioxidants found throughout the kingdom Plantae. It is known to play a central role in regulating the cellular redox potential in cells [102,103]. As humans and some other primates lack the ability to synthesize and store vitamin C, they depend on fresh fruits and vegetables to cover their daily requirements (75–90 mg RDA). All recent studies point toward a diet rich in vitamin C for improving human health. Troesch et al. [104], suggest that vitamin C should be a clear target for the nutritional enhancement of horticultural crops. The accumulation of vitamin C within the same species may vary between different cultivars [103,105,106], tissue types [107], and developmental stages [106,107]. Regardless of this variability, vitamin C is tightly regulated through net biosynthesis, recycling, degradation/oxidation, and/or intercellular and intracellular transport.

### 6.1. Occurrence of Ascorbic Acid

There are many authors reporting on differences between carrot cultivars regarding the content of vitamin C [108,109,110]. According to Matějková and Petříková [111], vitamin C content in six carrot cultivars ranged from 54 mg/kg to 132 mg/kg, while concentrations as low as 21 mg/kg [112] and high as 775 mg/kg were reported [109]. Vitamin C may accumulate at up to 20 mM in chloroplasts, and occurs in almost all parts of the cell. Dark orange carrots contain 4 times more vitamin C than yellow, purple and orange carrots [62].

### 6.2. Biosynthesis of Ascorbic Acid

In plants, four alternative pathways for ascorbic acid biosynthesis have been reported; namely, the d-mannose/l-galactose (d-Man/l-Gal) pathway, myoinositol pathway, galacturonate pathway, and l-glucose pathway. The ten-step d-Man/l-Gal pathway is the most acceptable for ascorbic acid biosynthesis in carrots (Figure 7). d-Glucose-6-phosphate (d-glucose-6-P), obtained from the hexokinase of d-glucose, is converted into its furanosyl derivative d-fructose-6-phosphate (d-fructose-6-P) in the presence of phosphoglucose isomerase (PGI). Phosphomannose isomerase (PMI) converts d-fructose-6-P into d-mannose-6-phosphate (d-mannose-6-P). d-Mannose-6-P subsequently rearranges (phosphate moves from C6 to C1) due to the catalytic action of phosphomannose mutase (PMM) to yield d-mannose-1-phosphate (d-Mannose-1-P).

d-Mannose-1-P is converted into glucose diphosphate d-mannose (GDP-d-mannose) in the presence of GDP-d-mannose pyrophosphorylase (GMP). GDP-d-Mannose undergoes a reversible reaction catalysed by GDP-d-mannose-3′,5′-epimerase (GME), and the unstable intermediate GDP-l-glucose is then readily converted into its isomer, GDP-l-galactose. GDP-l-galactose phosphorylase (GGP) converts GDP-l-galactose into l-galactose-1-phosphate (l-galactose-1-P), followed by dephosphorylation via the catalytic action of l-galactose-1-P phosphatase (GPP) to afford l-galactose. l-Galactose is converted into 1-galactono-1,4-lactone in the presence of l-galactose dehydrogenase (GalDH), which is dehydrogenized by l-galactono-1,4-lactone dehydrogenase (GalLDH) to yield l-ascorbic acid [113].

### 6.3. Factors Affecting Ascorbic Acid Concentration

Numerous factors affect the concentration of ascorbic acid in carrots, such as cultivar, carbon dioxide, temperature, processing, and storage.

#### 6.3.1. Cultivar

Nicolle et al. [62] studied the effect of genetic variability on vitamin C in 20 different carrot genotypes and observed significant differences. The concentration of vitamin C was highest in dark orange (four times), yellow (3.7 times), and white (2.3 times) carrot cultivars compared to the orange carrot cultivar. It has been reported that boron deficiency during the growth of carrots enhances ascorbic acid contents from 45% to 70% [26]. Ascorbic acid oxidase affects the stability of vitamin C in carrots. It converts l-ascorbic acid (active form of vitamin C) into dehydro-l-ascorbic acid via oxidation. Its affinity for l-ascorbic acid varies from 50 to 244 μM for different carrot genotypes [114,115]. Leong et al. [116] used a pulsing electric field to reduce ascorbic acid oxidase activity (thermo-stability) in Nantes, Solar Yellow, White Belgian, Nutri Red, and Purple Haze cultivars and calculated the catalytic activity of ascorbic acid through the Michaelis–Menten enzyme kinetic model. The range of V*_max_* values for studied genotypes was 9.54 to 34.71 μmol/min. The V*_max_* values for catalytic activity of ascorbic acid in white and yellow carrots were significantly (*p* < 0.05) higher than purple, red, and Nantes carrot genotypes. A pulsing electric field treatment of 0.8 kV/cm and 30 KJ/kg reduced variability of the thermo-stability of ascorbic acid oxidase in the puree of all studied genotypes.

#### 6.3.2. Elevated Carbon Dioxide

Carbon dioxide is important for photosynthesis in plants and affects vitamin C concentration. For example, recently Wu et al. [117] studied the consequence of elevated CO_2_ (3000 μmol/mol) on vitamin C accumulation in carrots. They concluded that elevated levels of CO_2_ significantly affected vitamin C accumulation due to the change in the transcript profile of 12 genes responsible for biosynthesis of vitamin C.

#### 6.3.3. Storage and Temperature

Vitamin C is sensitive to adverse handling. The level of vitamin C in baby carrots was reduced during cold storage in high and moderate O_2_ conditions; however, under a low O_2_ atmosphere, baby carrots retained the highest amount of vitamin C [27]. Frozen storage lessened vitamin C concentration by 4.1% [118]. The effect of prolonged storage was considerable losses of vitamin C, 15% to 49% [111,119]. After eight days of storage, vitamin C concentrations decreased by 38% at 7.5 to 8.5 °C and by 70% at 22 to 37.5 °C. The maximum decrease in vitamin C contents was observed during local storage at 25 to 28 °C [57]. Leong and Oey [114] reported that the thermal treatment (>80 °C, 10 min) before matrix destruction effectively inactivates ascorbic acid oxidase activity and protects l-ascorbic acid against enzymatic oxidation, consequently enhancing vitamin C concentration and stability in carrots.

#### 6.3.4. Processing

Thermal processing of carrots decreases the vitamin C concentration, while chemical preservatives, such as potassium bisulphate, aid in preserving vitamin C [120]. Conventional blanching enhances vitamin C contents from 37.5% to 85%, while ultrasound at temperatures above 60 °C has a negative effect on vitamin C [121]. Patras et al. [37] reported that blanched frozen samples of carrots have a higher vitamin C content compared to un-blanched frozen samples. Microwave assisted freeze dried carrots retain higher contents of l-ascorbic acid [122]. Vishwanathan et al. [123] investigated the effect of infrared assisted dry blanching and hybrid drying on carrots and concluded that infrared blanched-hybrid dried slices of carrots have higher (39%) vitamin C concentrations than water blanched-hot air-dried samples of carrots. Fast diffusion of air into the sample surface due to infrared heating and the removal of moisture at the same time makes the drying process faster.

### 6.4. The Health Benefits of Ascorbic Acid

Vitamin C (l-ascorbic acid) plays an important role in the biosynthesis of collagen, is essential for the synthesis of carnitine and catecholamines, and is also involved in the metabolism of cholesterol to bile acids. Vitamin C in an aqueous solution readily scavenges reactive oxygen and nitrogen species, and is part of the antioxidant network of the body. It plays a vital role in Fe absorption from the gut by reducing Fe^3+^ to Fe^2+^ and maintains the structure of Fe-binding proteins.

Vitamin C is involved in the regulation of hypoxia-inducible factor 1α (HIF 1α, a transcription factor that activates genes that control several mechanisms at the cell level, like cell survival, the development of new blood vessels, Fe transport, and glycolysis), which induces cellular responses to hypoxic conditions. It can aid in the treatment of neurodegenerative diseases like Alzheimer’s disease, Huntington’s disease, ischemic stroke, and Parkinson’s disease [124,125,126]. At high concentrations, it acts as a prodrug, and transports a high flux of H_2_O_2_ to cancer cells and plays a role in the treatment of cancer [127].

Scurvy, characterised by symptoms related to connective tissue defects, can be prevented with an adequate intake of l-ascorbic acid. Vitamin C maintains healthy skin, gums, and blood vessels. It also aids in the reduction of plasma cholesterol, the vitality of the immune system, and the elimination of reactive oxygen species. Leong and Oey [114] and Dias [128], also described detailed evidence on health benefits of vitamin C regarding its assistance against cancer, arteriosclerosis, and other cardiovascular diseases.

## 7. Conclusions and Future Challenges

It is evident from the present review that there is an abundant diversity of carrot cultivars grown successfully worldwide, delivering high agricultural yields. Due to the rich source of phytochemicals present in carrots, they serves as a multi-nutritional food source. The biological activities of some of the phytochemicals found in carrots; namely, phenolic compounds (particularly chlorogenic acid), carotenoids, polyacetylenes, and ascorbic acid (vitamin C), have indicated their potential to improve human health due to their anticancer, antioxidant, anti-inflammatory, antibacterial, plasma lipid modification, and serotogenic effects.

However, the concentration and nature of phytochemicals are affected by several factors, such as carrot genotype (colour differences), environmental conditions, and the preparation and storage of carrot products. Experiments addressing these factors are of great importance to improve the quality of carrots, and to develop genotypes enriched for selected beneficial phytochemicals.

Large quantities of carrots are annually discarded in different parts of the world because they do not meet market standards. Additionally, the carrot-processing industry (puree and juice) gives rise to a number of waste products, such as carrot peel, that can be recovered and used as a source of bioactive compounds. Thus, a series of valuable by-products, such as carotenoids, phenolic compounds, fractions of dietary fibre, and bioethanol, can be obtained from food-processing wastes and discarded carrots [6,129]. In addition, carrots can be processed for the production of anthocyanin-rich concentrate for pigment industry, while the resulting pomace can be extracted to obtain high-value-added phenolic compounds that can be used as functional food ingredients [130].
